# Self-Assembly of Porcine Parvovirus Virus-like Particles and Their Application in Serological Assay

**DOI:** 10.3390/v14081828

**Published:** 2022-08-20

**Authors:** Yanfei Gao, Haiwei Wang, Shanghui Wang, Mingxia Sun, Zheng Fang, Xinran Liu, Xuehui Cai, Yabin Tu

**Affiliations:** 1 State Key Laboratory of Veterinary Biotechnology, Harbin Veterinary Research Institute, Chinese Academy of Agricultural Sciences, Harbin 150069, China; 2 Regeneron Pharmaceuticals Inc., 777 Old Saw Mill River Road, Tarrytown, New York, NY 10591, USA

**Keywords:** porcine parvovirus, virus-like particles, diagnostic, I-ELISA

## Abstract

Porcine parvovirus (PPV) is widely prevalent in pig farms. PPV is closely related to porcine respiratory disease complex (PRDC) and porcine circovirus disease (PCVD), which seriously threatens the healthy development of the pig industry. Although commercial antibody detection kits are available, they are expensive and unsuitable for large-scale clinical practice. Here, a soluble VP2 protein of PPV is efficiently expressed in the *E. coli* expression system. The VP2 protein can be self-assembled into virus-like particles (VLPs) in vitro. After multiple steps of chromatography purification, PPV-VLPs with a purity of about 95% were obtained. An indirect, enzyme-linked immunosorbent assay (I-ELISA), comparable to a commercial PPV kit, was developed based on the purified PPV-VLPs and was used to detect 487 clinical pig serum samples. The results showed that the I-ELISA is a simple, cost-effective, and efficient method for the diagnosis of clinical pig serum and plasma samples. In summary, high-purity, tag-free PPV-VLPs were prepared, and the established VLP-based I-ELISA is of great significance for the sero-monitoring of antibodies against PPV.

## 1. Introduction

Porcine parvovirus (PPV), the common causative agent of reproductive failure associated with swine, belongs to the genus *Parvovirus* within the family *Parvoviridae*. The genotypes of PPV have been characterized to date, including seven strains from classical PPV type 1 (PPV1) to six novel strains (PPV2–PPV7). Among the seven viruses, PPV1, first discovered in cell culture contaminants in Germany in 1965 [1], is the most prevalent and is considered to be one of the main pathogens causing infertility and abortion in sows [2]. PPV2–PPV7 were discovered successively through detection techniques, such as metagenomic sequencing [3,4,5,6,7,8]. A PPV capsid is a small, non-enveloped, icosahedral, and spherical shell, with a diameter size of about 20~25 nm [9], that contains a single-stranded, linear DNA with a genome size of about 5~6 kilobases (kb) [10,11]. There are two main open reading frames (ORF) in the genome of PPV: ORF1, located at the 5′ end, encodes three nonstructural proteins (NS1, NS2, and NS3); and ORF2, located at the 3′ end, encodes three structural proteins (VP1, VP2, and VP3), among which VP2 is the main capsid component and the protective antigen [12].

PPV disease is widespread and mainly affects fetal pigs. PPV can cross the placental barrier to the fetus, which causes porcine reproductive disorder. In pig farms, sows often have clinical manifestations, such as mummification, reduced litter size, sow dystocia, and repeated mating, suggesting that the disease may break out in pig herds [13]. The disease mostly occurs in the spring, summer, sow parturition, and mating season, and once infected, it will quickly spread to the whole herd, making it challenging to eradicate. When sows are infected in the early stage of pregnancy, the mortality rate of embryos and fetuses can be as high as 80% to 100%. The most susceptible period is the first 30 to 40 days of pregnancy. If infection occurs during this period, fetuses become mummified, or smaller litters should be expected, resulting in substantial economic consequences [14]. If PPV infection occurs after fetal immunity has been acquired, fetuses become subclinical.

Virus-like particles (VLPs) are built from one or more units of viral capsid proteins by self-assembling them into morphological structures, with surface conformations similar to those of natural virus particles. Since VLPs do not contain viral nucleic acid, and their conformational epitopes imitate natural virus particles, VLPs are well known for many biological applications in disease control and prevention and diagnostic virology. For example, a VLP-based vaccine can offer several advantages over traditional vaccine approaches (inactivated whole viruses or attenuated viruses) based on a safe production process, low cost, high yield, a variety of overexpression systems, and robust immune responses. Now, PPV-VLPs can be constructed by genetic engineering, which is of great significance for the prevention of PPV. PPV-VLPs are composed of only the structural protein VP2s that automatically form a structure similar to naïve PPV virions in vitro, thus eliciting protective neutralizing antibodies when inoculated in pigs. Previous studies have shown that the recombinant PPV-VP2 protein can be successfully expressed in insect/baculovirus, yeast, and *E.coil* expression systems [15,16,17,18] and retains high immunogenicity.

The other application of VLPs is in the development of serological assays detecting antibodies against their natural pathogens. A high-throughput and reliable, serological assay with high sensitivity and specificity are essential for identifying the infected population and current seroprevalence. VLP-based serological assay naturally benefits from the high specificity of VLPs, thus minimizing cross-activity against other viruses. Serological ELISA coated with VLPs has been widely used to detect antibodies or neutralization epitopes [19,20]. For example, the ELISAs coated with VLPs of porcine circovirus type 2 (PCV2) and porcine circovirus type 3 (PCV3) have been reported and proved to have good sensitivity, specificity, and repeatability [21,22,23]. Recently, a commercial ELISA using an indirect ELISA (I-ELISA) format was developed to detect PPV1 with an unknown antigen expressed in baculovirus growth in insect cells. It is of great interest to compare this commercial kit with I-ELISA based on PPV1 VLPs expressed in *E. coli*. The *E. coli* expression system has the advantages of being cost-effective, having high growth rate, being easy to scale up, having reduced downstream bioprocesses, and having improved protein quality. The PPV-VLP-based I-ELISA (PPV-VLP-ELISA) assay developed based on the *E. coli* expression system should be ideal for extensively and dynamically monitoring PPV spread in swine herds. Considering there is only one serotype of PPV, the method is expected to detect antibodies against all genotypes. Furthermore, high cost and delayed delivery associated with importing currently available PPV commercial kits in the context of persistent inflation necessitate the need to develop domestic kits for efficient PPV control.

In this study, we described a proof-of-concept study to develop a serological I-ELISA method using recombinant PPV-VLPs expressed in the *E. coli* expression system and purified without the introduction of any fusion tags. The sensitivity and specificity of this I-ELISA assay were characterized. Agreement to the commercial I-ELISA kit and the prevalence of PPV in clinical pig samples using this I-ELISA were investigated.

## 2. Materials and Methods

### 2.1. Swine Serum Samples

To ensure the specificity of the assay, serum samples were screened for only PPV infection, excluding other pathogens, such as porcine reproductive and respiratory syndrome virus (PRRSV), pseudorabies virus (PRV), and PCV2 by ELISA and PCR. The samples with the highest concentration were selected as positive serum and employed to coat ELISA plates for the PPV-VLP-ELISA and used for ELISA optimization and Western blot. Negative serum samples were obtained from 55 specified, pathogen-free (SPF) piglets, which were collected from the Experimental Animal Center at the Veterinary Research Institute (Harbin, China). A total of 487 clinical serum samples were collected from northeastern China in 2020 and 2021 for testing via the PPV-VLP-ELISA.

### 2.2. Gene Amplification and Optimization

The VP2 sequence of PPV obtained from Genbank (Accession No. MF447833) was used as a reference. Subsequently, the VP2 gene was codon-optimized and synthesized by the Genscript Corporation and ligated into the expression vector pET28a.

### 2.3. Construction and Expression of Recombinant VP2 Protein in E. coli

The recombinant vector pET28a-PPV-VP2 was transformed into BL21(DE3)-competent cells containing chaperone pTf-16. Monoclonal bacteria were selected on the plate containing kanamycin and chloramphenicol, then activated for 12 h at 37 °C and 220 rpm/min in 5 mL of TB medium, which contained 160 ug/mL of chloramphenicol and 50 ug/mL kanamycin. Subsequently, 4 mL of bacterial solution was transferred to 200 mL of TB culture medium at the ratio of 1:50 at the same temperature and shaking speed for 2 h 30 min, after which the temperature was reduced to 16 °C, and IPTG and L-Arabinose with the final concentration of 0.1 mmol/L and 2 mg/ mL were added to induce VP2 expression for 20 h. Upon completion of protein induction, the bacteria culture was centrifuged at 6000 g/min for 10 min. The cell pellets were weighed (g) and resuspended in a disruption buffer (200 mM NaCl, 20 mM Tris-HCl, 10% glycerol, pH 8.0) at a ratio of 1 (wet bacteria weight (g)): 10 (disruption buffer (mL)). After mixing well, sonication was performed with an ultrasonic cell disruptor (Cole Parmer, Vernon Hills, IL, USA). SDS-PAGE and Western blot were used to check the expression and solubility of the recombinant VP2 protein. After protein electrophoresis, one part was stained with Coomassie brilliant blue, and the other part was transferred to a PVDF membrane in PBST for 2 h. The PVDF membrane was washed three times with PBST for 15 min and probed with the PPV-specific positive serum (1:5000 dilution) for 1 h at RT. The PVDF membrane was washed three times again and detected with fluorescently-labeled anti-pig secondary antibody (Biodragon, Beijing China, 1:10,000) for 40 min at RT. The PVDF membrane was then washed three times again in the dark and then scanned on a near-infrared fluorescence scanning imaging system (Odyssey CLX, USA).

### 2.4. Purification of PPV-VLPs

The sonicated solution was clarified at 12,000 g/min for 30 min to remove bacterial debris and inclusion bodies. The supernatant was precipitated with PEG6000, and the protein precipitate was centrifuged at 12,000 g/min for 30 min. The pellet was resuspended in resuspension buffer (20 mM Tris, pH 8.0). The resuspended solution was loaded on a DEAE Bestarose Fast Flow column (Bestchrom, Shanghai, China) in an automated FPLC system (AKTA, GE-Healthcare Life Sciences, USA), and the fractions containing effluent from the bulk VLPs were collected. The effluent fractions were subjected to a Sepharose 6FF 16/96 column (Bestchrom, China) equilibrated with equilibration buffer (20 mM Tris-HCl, pH 8.0) at the flow rate of 1.5 mL/min, and the second half of the first peak was collected. The collected protein fractions were further loaded onto a heparin-agarose column (Bestchrom, China) and eluted with elution buffer (500 mM NaCl, 20 mM Tris-HCl). Finally, the eluted fraction was passed through a Sepharose 6FF 16/96 column (Bestchrom, China), which was equilibrated with equilibration buffer (20 mM Tris-HCl, pH 8.0) at a flow rate of 1.5 mL/min. The first peak was collected in separate 15 mL centrifuge tubes, with 10 mL per tube. The protein elutions were analyzed with SDS-PAGE and TEM.

### 2.5. TEM Procedure of PPV-VLPs

The sample was incubated for 10 min at RT. Subsequently, the PPV-VLPs were fully adsorbed onto the copper mesh and dried at RT. The copper mesh was negatively stained with 2.5% phosphotungstic acid for 1 min after being dried, and the excess stain was blotted off using filter paper and carefully placed on the TEM (HITACHI, Tokyo, Japan) for observation.

### 2.6. Determination of Hemagglutination of PPV-VLPs

The hemagglutination activity of PPV-VLPs was determined by a hemagglutination assay. In a 96-well V-plate, 25 uL PBS was added to each well, then 25 uL PPV-VLPs were added to the first column of wells and mixed to generate 2-fold dilution. Then, 25 uL of the 2-fold-diluted PPV-VLPs were added to the second column of wells and mixed, repeating the dilution pattern across the plate to complete the 2-fold serial dilutions of antigen PPV-VLPs. A total of 25 uL 1% chicken erythrocyte suspension was added to the plate and shaken for 2 min to mix well. The control group used only 25 uL of PBS and 25 uL of 1% chicken red blood cell suspension. The results were determined 1 h later, and the highest dilution that allowed 100% agglutination of red blood cells was the hemagglutination titer of PPV-VLPs.

### 2.7. Optimization of the PPV-VLP-ELISA Procedure

Purified PPV-VLPs were used as antigens to develop I-ELISA. The checkerboard method was applied to determine the optimal serum dilution and antigen-coating concentration. The concentration of PPV-VLPs was determined by a BCA kit (Thermo, Waltham, MA, USA). PPV-VLPs were diluted to 0.5 ug/mL, 1 ug/mL, 2.5 ug/mL, 5 ug/mL, 7.5 ug/mL, and 10 ug/mL in carbonate coating buffer (pH 9.6) and then plated on ELISA plates (Biofil, Guangzhou, China) to determine the optimal antigen-coating concentration. Positive and negative sera were diluted at 1:50, 1:100, and 1:150 (*v*/*v*) with PBST to determine the optimal serum dilution. Additionally, the reaction time, temperature, and other conditions were optimized.

### 2.8. Standardization of PPV-VLP-ELISA Procedure

PPV-VLPs were coated with 100 μL/well, at a concentration of 1 μg/mL, in a 96-well ELISA microplate (Biofil, China) overnight at 4 °C. The coated plates were washed three times with PBST and then blocked with an enzyme plate stabilizer (InnoReagents, China) for 2 h in a 37 °C incubator (Yiheng, Shanghai, China). The blocked plates were washed three times, and 100 μL of the diluted serum samples were added and incubated at 37 °C for 1 h. Then, the plates were washed three times, and 100 μL HRP-SPA dilution (Bosterbio, Wuhan, China, 1:10,000) was added and incubated for 1 h at 37 °C. After the three-time washes, 100 μL of tetramethylbenzidine (TMB, Solarbio, Beijing, China) was added to each well and incubated for 15 min at 37 °C in the dark. Finally, 50 μL of stop solution (1M HCl) was added to stop the reaction, and the OD450nm value was measured with an ELISA plate reader (PE, Corona, CA, USA).

### 2.9. Determination of Cut-Off Value

Fifty-five negative sera were employed to determine the cut-off value. All sera were analyzed by PPV-VLP-ELISA three times independently to reduce the deviation. The mean OD450nm value (X) and standard deviation (SD) were calculated, and the cut-off value was defined as X + 3SD.

### 2.10. Reproducibility and Cross-Reactivity Assay

Twelve serum samples were selected to evaluate the reproducibility of the PPV-VLP-ELISA. For each sample, the coefficient of variation (CV) values between plates (inter-assay variation) and within plates (intra-assay variation) were calculated. The results showed that both the inter-assay and intra-assay variation were less than 10%. Four positive sera and one negative serum diluted at 1:50, 1:100, 1:200, 1:400, 1:800, 1:1600, 1:3200, and 1:6400 were applied to evaluate the sensitivity of this method. The specificity of the method was evaluated by comparing the OD450nm values of the standard positive serum with those of the selected, potentially interfering viruses (African swine fever virus (ASFV), Japanese encephalitis virus (JEV), PRRSV, foot-and-mouth disease virus (FMDV), PCV2, simian immunodeficiency virus (SIV), transmissible gastroenteritis virus (TGEV), and porcine epidemic diarrhea virus (PEDV)).

### 2.11. Cultivation and Proliferation of PPV

Pig kidney (PK-15) cells were obtained and preserved by the Harbin Veterinary Research Institute (Harbin, China). The PK-15 cells were revived in the cell culture flask, and when the bottom of the flask was confluent with PK-15 cells, the cells were digested and transferred to a new flask. PPV was then inoculated into the flask and cultured in a cell incubator (Thermo, USA) at 37 °C and 5% carbon dioxide. When about 80% of the inoculated cells became cytopathic, they were repeatedly incubated. The viruses were fully released by three freeze–thaw cycles and were harvested at 2000 r/min for 10 min to remove cell debris and frozen at −80 °C.

### 2.12. Comparison of the PPV-VLP-ELISA with the Commercial PPV ELISA Kit for Detection of Anti-PPV Antibodies

Sixty-four samples were randomly selected from 487 serum samples for comparison between commercial ELISA kits (Ingenasa, Spain) and PPV-VLP-ELISA. Sixty-four samples were tested using a commercial kit, according to the instructions, and positive and negative samples were placed and labeled separately. These samples were subsequently tested using PPV-VLP-ELISA. Each serum was analyzed three times independently, using commercial kits and PPV-VLP-ELISA to minimize bias.

### 2.13. Indirect Immunofluorescence Assay Verification of Five Positive Samples Determined by PPV-VLP-ELISA

The five positive serum samples to be tested were named A, B, C, D, and E, respectively, and then an indirect immunofluorescence assay (IFA) was performed. First, PK-15 cells were infected with PPV for 48 h, after which the cells were fixed with 4% paraformaldehyde for 30 min at RT and then washed with PBS three times. Permeabilization fluid (0.25% Triton-X100) was added at 100 uL/well and was placed on ice for 15 min. After washing three times with PBS, a blocking solution (5% BSA) was added for 1 h. After another three-time wash, the serum samples and SPF serum were diluted at 1:100, added to the wells, and incubated at 37 °C for 2 h. After another three-time wash, a FITC-labeled goat anti-pig secondary antibody (Sigma, Burlington, MA, USA) was added at a dilution of 1:200 and incubated at 37 °C for 1 h. After the last three-time washes, results were measured with an inverted fluorescence microscope (AMG, Denver, CO, USA).

## 3. Results

### 3.1. Expression and Purification of PPV-VLPs from E. coli

**Figure 1:**
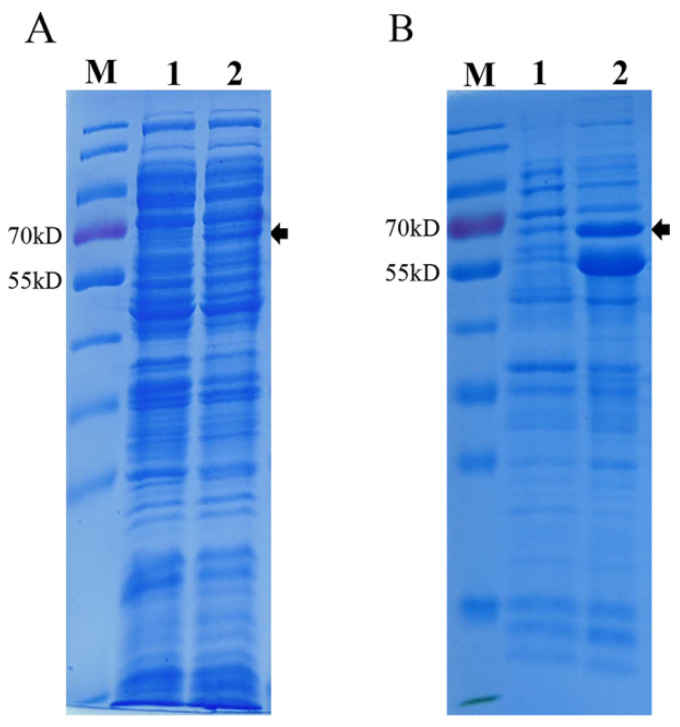
SDS-PAGE analysis of PPV VP2 protein expression in E. coli. (**A**) Coomassie staining of PPV VP2 protein expressed in *E. coli* without chaperone pTf-16. (**B**) Coomassie staining of PPV VP2 protein in *E. coli* co-expressed with chaperone pTf-16. M. Marker. Lane 1, Coomassie staining of PPV VP2 protein before IPTG and L-Arabinose induction. Lane 2, Coomassie staining of PPV VP2 protein after IPTG and L-Arabinose induction.

**Figure 2:**
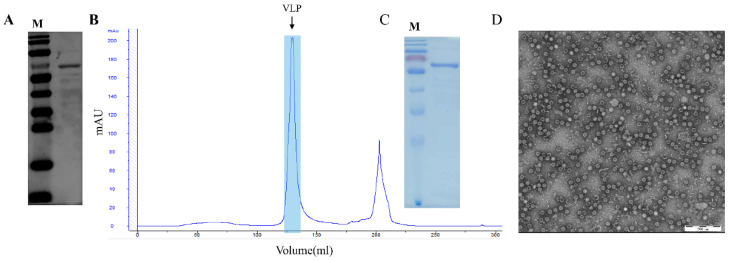
Analysis and purification of PPV VP2 protein and PPV-VLPs after initial HP. (**A**) Western blot analysis of PPV VP2 protein probed with specific positive serum. (**B**) Heparin-affinity profile of self-assembled PPV-VLPs. The peak represents properly-assembled PPV-VLPs. (**C**) Coomassie staining of the PPV VP2 protein. (**D**) The TEM image of the PPV-VLPs.

**Figure 3:**
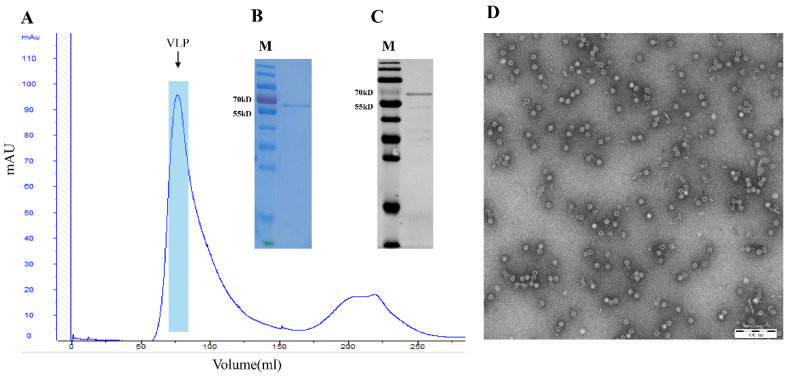
Analysis of PPV VP2 protein and PPV-VLPs after 2nd SEC purification. (**A**) SEC profile of PPV-VLPs. The peak represents properly-assembled PPV-VLPs. (**B**) Coomassie staining of the PPV VP2 proteins after 2nd SEC purification. (**C**) Western blot analysis of the more purified PPV VP2 proteins. (**D**) TEM image of the PPV-VLPs.

The recombinant plasmid pET28a-PPV-VP2 was constructed and transformed in *E. coli* BL21 (DE3) competent cells, and cells containing the plasmid were inoculated in TB medium. The recombinant VP2 protein (molecular weight ~64 kDa) was IPTG- and L-Arabinose-induced with or without the co-expression of the chaperone plasmid pTf-16. When expressed in the absence of chaperone pTf-16, the VP2 protein was poorly expressed and less soluble (Figure 1A). When co-expressed with chaperone pTf-16, both the expression and solubility of the VP2 protein significantly increased (Figure 1B). Western blot analysis also confirmed the folding of the VP2 protein obtained from chaperone pTf-16 co-expression, as it reacted with specific positive sera (Figure 2A). As described above, expressed VP2 proteins were purified by three columns: ion-exchange column (IEC), size-exclusion column (SEC), and heparin-agarose column (HP). While self-assembled PPV-VLPs were observed after HP (Figure 2B), a few heterogeneous protein bands and odd-shaped impurity particles around 25 nm could also be clearly observed under SDS-PAGE (Figure 2C) and TEM (Figure 2D). PPV-VP2 proteins were further purified via another SEC (Figure 3A–C). Homogeneous and intact particles without any impurities were observed under TEM (Figure 3D).

### 3.2. Hemagglutination Activity of PPV-VLPs

**Figure 4:**
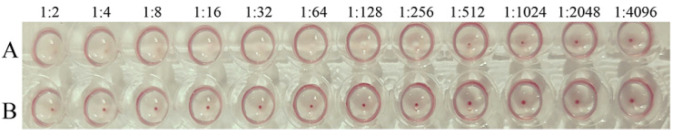
The hemagglutination test of PPV-VLPs. (**A**) Hemagglutination titer of the purified PPV-VLPs. (**B**) The negative control.

The hemagglutination activity of PPV-VLPs was confirmed to be 2^8^ (1:256) (Figure 4), indicating that the PPV-VLPs correctly displayed the epitope of PPV required for hemagglutination activity, and the structure was similar to that of the native PPV.

### 3.3. Standardization of the PPV-VLP-ELISA Procedure

**Figure 5:**
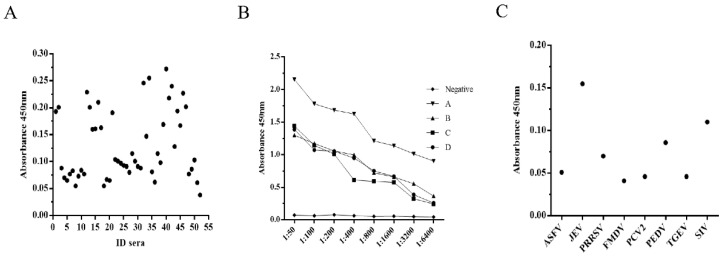
Characterization of PPV-VLP-ELISA. (**A**) Determination of the cut-off value for PPV-VLP-ELISA. (**B**) Determination of the sensitivity of PPV-VLP-ELISA. (**C**) Determination of the specificity of PPV-VLP-ELISA.

The optimization of the VLP-based I-ELISA was guided by OD450nm and P/N values (ratio of the OD value of a test sample to the average OD value of negative control). A total of 50 mM of carbonate-bicarbonate buffer (pH 9.6) and plate stabilizer were selected as the final coating and blocking buffers, respectively. The concentration of purified PPV-VLPs was determined to be 0.1 mg/mL using the BCA protein concentration measurement kit (Thermo, USA). Checkerboard titration experiments showed that when coating concentration for PPV-VLPs was 1 μg/mL and the serum dilution was 1:50, the P/N ratio can be ultimately optimized. All experiments were performed in triplicate (Table 1). Fifty-five negative serum samples were used to determine cut-off values, with the mean OD and SD values of 0.149 and 0.059, respectively (Figure 5A). Therefore, the cut-off value for the PPV-VLP-ELISA was 0.326 (X + 3SD). Serum with an OD450nm value greater than or equal to this threshold was considered positive. Otherwise, it was determined to be negative for PPV antibodies. The experiments showed that this optimized method has good sensitivity and specificity (Figure 5B,C).

### 3.4. Coincidence Rate with the Ingezim PPV ELISA Kit for Detection of Anti-PPV Antibodies

Clinical pig serum samples (*n* = 64) were used to determine the reliability of the PPV-VLP-ELISA compared to the commercial PPV-ELISA kit. These sera were randomly selected from 487 serum samples and tested using both commercial ELISA kits and PPV-VLP-ELISA developed in-house. When tested using the commercial ELISA kit, 36 (56.25%) of 64 serum samples were positive and 28 (43.75%) of 64 serum samples were negative. When tested using the PPV-VLP-ELISA method, 41 (64.1%) of 64 serum samples were positive and 23 (35.9%) of 64 serum samples were negative (Table 2). The results were similar to the commercial ELISA kit, as the overall concordance rate was 92.2% (59/64) between PPV-VLP-ELISA and the commercially available PPV-Ingenasa-ELISA kit. Among 41 samples tested positive by PPV-VLP-ELISA, 36 samples also tested positive in the commercial kit and the other 5 samples tested negative in the commercial kit. The results suggested that the sensitivity and specificity of PPV-VLP-ELISA were at least comparable to those of the commercial kits.

### 3.5. Confirmation of PPV-VLP-ELISA Tested Positive Samples with Indirect Immunofluorescence Assay

**Figure 6:**
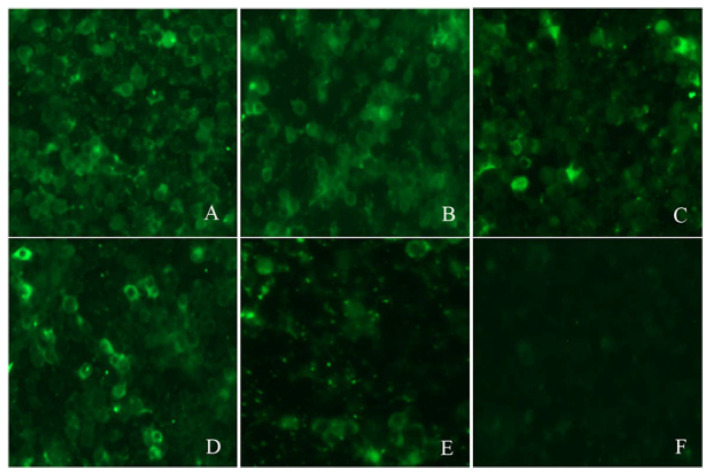
Reactivity of five positive sera with PPV antibodies by IFA. (**A**–**E**) are the IFA results of the five sera, respectively. (**F**) IFA analysis of SPF pig serum (negative control).

The IFA results agreed with the PPV-VLP-ELISA results. Five positive sera determined by PPV-VLP-ELISA were also confirmed positive by IFA. SPF serum as a negative control was proved to be negative by IFA (Figure 6).

### 3.6. Application of PPV-VLP-ELISA to Screen Clinical Pig Serum Samples

**Figure 7:**
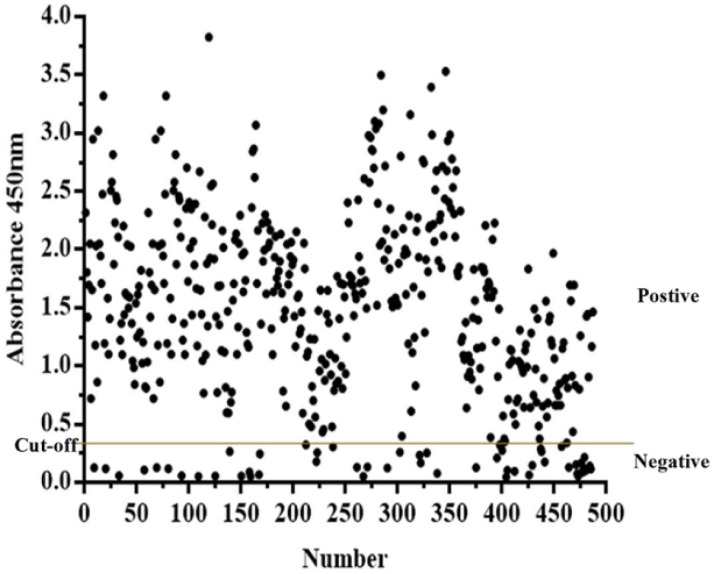
PPV-VLP-ELISA was employed to screen clinical swine serum samples (*n* = 487).

Swine serum samples collected in northeast China in 2020 and 2021 were tested with PPV-VLP-ELISA. A total of 432 samples (88.7%) tested positive, and 55 samples (11.3%) tested negative. The result showed a high positive rate of PPV antibodies in the tested herd (Figure 7), indicating that PPV is widespread in pig farms and poses a serious threat to the local pig industry. In conclusion, PPV-VLP-ELISA has high application value in the monitoring and eradication of PPV in the future.

## 4. Discussion

Currently, different expression host systems can express VLPs, including bacteria, insect, yeast, mammalian cell, cell-free, and plant expression systems [24]. Recombivax HB, the world’s first genetically engineered vaccine for the prevention of hepatitis B, is a VLP vaccine expressed by the *saccharomyces cerevisiae* expression system [25,26]. From then on, multiple VLP vaccines were approved, including human papillomavirus (HPV), hepatitis B virus (HBV), hepatitis E virus (HEV), and H1N1 vaccine, etc. [27]. About 30% of approved VLP vaccines are produced by bacterial expression systems, mainly in *E. coli*. Other VLP bacterial expression systems besides *E. coli*, *Lactobacillus*, and *Pseudomonas aeruginosa* are responsible for vaccines against HPV and cowpea chlorosis mottle virus (CCMV) [28,29]. While bacterial expression systems may have many advantages in expressing VLPs, such as high growth rate, being easy-to-scale up, and low cost, their applications should be evaluated cautiously due to the lack of post-translational modification of proteins. Concerns about endotoxin contamination may further reduce the scope of its application [30]. The yeast expression system is widely used to produce VLPs for HPV and HBV. Insect and mammalian cell expression systems have been used to produce VLPs since the 1980s. The plant cell expression system and cell-free expression system have recently been found capable of producing VLPs.

VLPs only contain viral capsid proteins but no viral genetic material. Their conformational epitopes and morphological structure are similar to those of natural viruses and have good immunogenicity [31,32]. VLPs can induce strong humoral and cellular immune responses and are considered a highly efficient vaccine platform [33]. In recent years, the technologies of expressing PPV VP2 proteins and self-assembly into VLPs have been greatly advanced. These new technologies have been advancing the production and future development of VLP-based vaccines [18]. PPV VP2 is a non-enveloped protein that can self-assemble into VLPs under suitable conditions, without additional modifications. Therefore, compared with other eukaryotic expression systems, the *E. coli* expression system, without protein modifications, is proper and desirable for producing VP2 proteins. Assembled PPV-VLPs are highly likely to form conformation similar to that of natural viral particles. The suitability of the *E. coli* expression system to produce the VP2 protein was verified by both the results from PPV-VLP-ELISA and previous studies regarding VLP-based vaccines [18,34].

In this study, the *E. coli* expression system has been employed successfully to express and purify tag-free PPV-VLPs. PPV-VLPs have many advantages, including cost-effectiveness, high-yield expression, and high-density cultivation. Thus, it is significant for diagnosis and vaccine development.

The VLP has only viral structural proteins without viral genetic material, and its morphological structure is similar to that of natural virus particles [35,36]. It is capable of mimicking external epitopes and conformational epitopes of viruses [37,38]. Therefore, the VLP is more suitable for establishing the ELISA diagnostic method. Recent studies have shown that the PPV is closely related to porcine respiratory diseases, so monitoring and vaccination against the PPV plays an important role in the prevention and treatment of PRDC and PCVD [39,40]. Therefore, establishing a diagnostic method with high sensitivity, high specificity, and suitability for large-scale screening is of great significance for the control of PPV. This paper successfully showed a high-sensitivity, high-specificity, and low-cost PPV-VLP-ELISA method using high-purity PPV-VLPs. Subsequently, the VLP-ELISA method was successfully used for PPV antibody detection in 487 clinical serum samples collected in northeast China in 2020 and 2021. It was found that PPV was widely prevalent in Chinese pig farms, indicating that this method can be widely used in PPV epidemiological studies.

## 5. Conclusions

The *E. coli* expression system has been employed for the expression and purification of tag-free PPV-VLPs, which could be used for the development of a VLP-based PPV vaccine. In addition, this is the first report of the I-ELISA method based on PPV-VLPs for testing the PPV-specific antibodies in clinical pig serum. The PPV-VLP-ELISA is highly specific, sensitive, and reproducible. It is a valuable tool for monitoring the prevalence of PPV.

## Figures and Tables

**Table 1:** Optimal sample dilutions and coating antigen for PPV-VLP-ELISA.

Serum Dilution	Concentration of Coating Antigen (X ± SD, ug/mL)					
	10	7.5	5	2.5	1	0.5
1:50 (+)	1.194 ± 0.0898	1.524 ± 0.0757	1.675 ± 0.0764	1.199 ± 0.0396	1.966 ± 0.0233	1.225 ± 0.0452
1:50 (−)	0.0985 ± 0.002	0.0965 ± 0.002	0.1565 ± 0.023	0.1085 ± 0.013	0.1225 ± 0.011	0.113 ± 0.01
P/N	13.13	15.78	10.70	11.05	17.47	10.84
1:100 (+)	0.517 ± 0.021	0.8335 ± 0.047	1.1095 ± 0.046	0.6395 ± 0.122	1.52 ± 0.1075	0.945 ± 0.006
1:100 (−)	0.1 ± 0.002	0.1185 ± 0.0021	0.149 ± 0.0028	0.0915 ± 0.009	0.119 ± 0.01	0.1105 ± 0.004
P/N	5.10	7.3	7.45	6.99	12.77	8.55
1:150 (+)	0.728 ± 0.11	1.156 ± 0.113	1.1011 ± 0.016	0.754 ± 0.0339	1.268 ± 0.012	0.8925 ± 0.0446
1:150 (−)	0.127 ± 0.037	0.087 ± 0.0212	0.0875 ± 0.013	0.174 ± 0.0368	0.087 ± 0.007	0.1185 ± 0.0629
P/N	5.73	13.29	11.55	5.26	12.22	6.69

**Table 2:** Comparison of the PPV-VLP-ELISA with the commercial PPV ELISA kit for detection of anti-PPV antibodies.

PPV-VLP-ELISA	Commercial Kit		
	Positive	Negative	Total
Positive	36	5	41
Negative	0	23	23
Total	36	28	64

## Data Availability

The data presented in this study are available upon request from the corresponding author.
